# Editorial: Interaction in robot-assistive elderly care

**DOI:** 10.3389/frobt.2022.1020103

**Published:** 2022-09-29

**Authors:** Hidenobu Sumioka, Jim Torresen, Masahiro Shiomi, Liang-Kung Chen, Atsushi Nakazawa

**Affiliations:** ^1^ Advanced Telecommunications Research Institute International, Seika, Japan; ^2^ Department of Informatics, University of Oslo, Oslo, Norway; ^3^ Institute of Neuroscience, National Yang Ming Chiao Tung University, Taipei, Taiwan; ^4^ Department of Intelligence Science and Technology, Graduate School of Informatics, Kyoto University, Kyoto, Japan

**Keywords:** elderly care robots, social robot, human-centered robotics, sensor systems for elderly care, human-robot interaction (HRI)

With the spread of COVID-19, elderly care is becoming an even more serious issue to which robots are expected to contribute. In addition to rehabilitation and physical support, researchers also pay attention to the application of social robots for mental support, such as preventing dementia and BPSD (Behavioral and Psychological Symptoms of Dementia) through social interaction. In elderly care, due to cognitive and socio-emotional decline and mental diseases, older people have difficulties interacting smoothly with their caregivers because their ways of interaction differ from what is common among healthy adults. Furthermore, since these effects vary widely among individuals, care should be customized to everyone’s needs, as suggested by person-centered care. However, current social robots cannot provide such care.

This Research Topic focuses on scientific and technical advances in methods, models, techniques, algorithms, and interaction design developed to understand and facilitate verbal and non-verbal interaction between older people and caregivers/artificial systems. In this collection containing seven peer-reviewed articles, the studies can be divided into two categories.

The first category contributes to developing robots to support older people, including those with dementia. Sumioka et al. propose to promote emotional interaction with older people with dementia by introducing an interactive baby robot. The robot is designed with only the minimum elements necessary to encourage the user’s imagination and does not even have a face. The interaction with the robot is also simple: it frequently laughs when a user physically engages it by lifting or rocking it, but it cries otherwise. Their results with older people with dementia show that even such a simple robot can elicit rich responses from them.

Otake-Matsuura et al. investigate the effect of Photo-Integrated Conversation Moderated by Robots (PICMOR), a group conversation intervention program for resilience against cognitive decline and dementia, on healthy older people. PICMOR contains discussing photos taken by each participant with all participants and assigning a robot as a moderator to coordinate their speaking time. They compare PICMOR with an unstructured group conversation with a single-site randomized controlled trial (RCT). Their 12-weeks interventions using PICMOR resulted in significantly improved verbal fluency. This study demonstrates the positive effects of robotic social activity interventions on cognitive function in healthy older people.

Tokunaga et al. explore a design method that uses a dialogue-based robot system for cognitive training at home. They first highlight three challenges to realizing such a system and propose three functional and non-functional requirements. Based on them, they developed a diagram for a dialogue-based system with photo and storytelling (DBSPS) as a testbed. They conducted three user studies in prototype- and laboratory- and home-based experiments to test their system. They discuss the system's essential characteristics to experiment with daily cognitive training.

One might wonder whether a physical embodiment is necessary to facilitate communication with older people. Nishio et al. address this question by comparing two physical robots with two virtual agents. They test two hypotheses: older people are 1) more engaged when the physical robots ask about their experience than when the virtual agents do and 2) feel virtual agents as closer than physical ones. Their results reveal the first hypothesis is valid. Older people engage more in the conversation with the physical robots than the virtual robots when robots successfully respond to them. However, the second hypothesis is not supported, suggesting that having a physical body is advantageous in promoting high engagement, depending on whether the system works well.

Moustris et al. point out the problem of locomotion among the elderly and developed a wheelbarrow robot called i-Walk, which offers cognitive and mobility assistance to the elderly and people with light to moderate mobility impairment. They evaluate the performance of the robot’s functions and users’ impressions of the robot in four scenarios. Their results show that patients and caregivers generally show a positive impression of the system, although some functions such as speech recognition should be improved.

The second category contributes to developing robots to support caregivers. The improvement of caregiving skills directly affects the QoL of the elderly. Lee et al. propose a care training assistant robot (CaTARo) with 3D facial pain expression that simulates an older person to improve caregivers’ skills in elderly care. Their system introduces a fuzzy logic–based care training evaluation method that can display a facial expression of a 3D facial avatar as the patient’s pain level in the system. Their feasibility study confirms the proposed approach has the potential to improve caregiving and nursing skills.

To support care in various situations, Krüger et al. propose the SMOOTH-robot, a mobile robot to assist humans in various care situations. The robot is designed to either make itself ready or be quickly changed by caregivers to perform different tasks. They demonstrate the potential of the SMOOTH-robot through three use cases, two of which were performed in elderly care homes. They show that the robot can help caregivers perform actual caregiving tasks. They also discuss the robot's application in other domains.

Understanding human care skills is also important for developing robots that support caregivers. Sumioka et al. overviewed care techniques in Humanitude, which provides caregivers with practical interaction skills to improve the relationships with persons with dementia (PwDs), for developing a social robot that can smoothly interact with PwDs. They first present the four crucial caregiving skills in Humanitude (face-to-face interaction, verbal communication, touch interaction, and helping care receivers stand up). Then, they point out that current social robots cannot achieve these caregiving skills and present several technical challenges to implementing Humanitude techniques in social robots.

Due to COVID-19, nursing homes need additional support: the burden on caregivers has increased due to infection prevention measures, and the isolation of the elderly with dementia due to the drastic decrease in social interaction has become an issue. An understanding of human skills for elderly care and the development of caregiving assistive technologies are needed to address these issues. This Research Topic may help realize new caregiving technologies that can improve the QoL of caregivers and older people. We hope you will find the selected papers of interest and that they represent a valuable contribution to the interaction in the robot-assistive elderly care research domain.

